# “The post-COVID era”: challenges in the treatment of substance use disorder (SUD) after the pandemic

**DOI:** 10.1186/s12916-020-01693-9

**Published:** 2020-07-31

**Authors:** Hugo López-Pelayo, Henri-Jean Aubin, Colin Drummond, Geert Dom, Francisco Pascual, Jürgen Rehm, Richard Saitz, Emanuele Scafato, Antoni Gual

**Affiliations:** 1 Grup Recerca Addiccions Clínic (GRAC-GRE), Institut d’Investigacions Biomèdiques August Pi I Sunyer (IDIBAPS), Hospital Clínic Barcelona, Rosselló 149, 08036 Barcelona, Spain; 2 Socidrogalcohol (Spanish Society of Drug and Alcohol Specialists), Barcelona, Spain; 3 Département de psychiatrie et d’addictologie, Université Paris-Saclay, Route de l’Orme aux Merisiers - RD 128 91190 Saint-Aubin, Paris, France; 4 Centre de Recherche en Epidémiologie et Santé des Populations (CESP), INSERM 1018, Paris, France; 5 Groupe Hospitalo-Universitaire AP-HP, Paris, France; 6 Addiction Psychiatry, Addictions Department, National Addiction Centre, Institute of Psychiatry, Psychology and Neuroscience King’s College, 16 De Crespigny Park, Camberwell, London, SE5 8AF UK; 7 European Federation of Addiction Societies (EUFAS), 16 De Crespigny Park, Camberwell, London, SE5 8AF UK; 8 Antwerp University (UA, CAPRI), Antwerp, Belgium; 9 Belgian Professional Psychiatry Association, Antwerp, Belgium; 10 European Federation of Addiction Societies (EUFAS), Antwerp, Belgium; 11 European Psychiatric Association (EPA), Prinsstraat 13, 2000 Antwerp, Belgium; 12 SOCIDROGALCOHOL, Barcelona, Spain; 13 CAARFE, Valencia, Spain; 14 Departamento de Biología Aplicada, Alicante, Spain; 15 UCA, Alcoy, Alicante Spain; 16 Institute for Mental Health Policy Research & Campbell Family Mental Health Research Institute, Centre for Addiction and Mental Health (CAMH), Toronto, Canada; 17 Dalla Lana School of Public Health and Department of Psychiatry, University of Toronto (UofT), 155 College St., Toronto, Canada; 18 Epidemiological Research Unit, Technische Universität Dresden, Klinische Psychologie and Psychotherapie, Dresden, Germany; 19 Department of International Health Projects, Institute for Leadership and Health Management, I.M. Sechenov First Moscow State Medical University, Moscow, Russian Federation; 20 Department of Community Health Sciences (CHS), Boston University School of Public Health, 715 Albany St, Boston, MA 02118 USA; 21 Medicine, Clinical Addiction Research and Education (CARE) Unit, Section of General Internal Medicine, Boston University School of Medicine, Boston, MA 02118 USA; 22 Grayken Center on Addiction, Boston Medical Center, Boston, MA 02118 USA; 23 WHO Collaborating Centre on Research and Health-Osservatorio Nazionale Alcol, Promotion on Alcohol and Alcohol-related Health, Problems (ITA-79), Istituto Superiore di Sanità, Rome, Italy; 24 Società Italiana di Alcologia – SIA, EUFAS Istituto Superiore di Sanità, Rome, Italy; 25 Grup Recerca Addiccions Clínic (GRAC-GRE), Institut d’Investigacions Biomèdiques August Pi I Sunyer (IDIBAPS), Hospital Clínic Barcelona, Rosselló 149, 08036 Barcelona, Spain; 26 International Network on Brief Interventions for Alcohol and Other Drugs (INEBRIA), Barcelona, Spain

**Keywords:** Addictions, COVID-19, Substance use disorder, Stigma, Telemedicine, Harm-reduction

## Abstract

**Background:**

Citizens affected by substance use disorders are high-risk populations for both SARS-CoV-2 infection and COVID-19-related mortality. Relevant vulnerabilities to COVID-19 in people who suffer substance use disorders are described in previous communications. The COVID-19 pandemic offers a unique opportunity to reshape and update addiction treatment networks.

**Main body:**

Renewed treatment systems should be based on these seven pillars: (1) telemedicine and digital solutions, (2) hospitalization at home, (3) consultation-liaison psychiatric and addiction services, (4) harm-reduction facilities, (5) person-centered care, (6) promote paid work to improve quality of life in people with substance use disorders, and (7) integrated addiction care. The three “best buys” of the World Health Organization (reduce availability, increase prices, and a ban on advertising) are still valid. Additionally, new strategies must be implemented to systematically deal with (a) fake news concerning legal and illegal drugs and (b) controversial scientific information.

**Conclusion:**

The heroin pandemic four decades ago was the last time that addiction treatment systems were updated in many western countries. A revised and modernized addiction treatment network must include improved access to care, facilitated where appropriate by technology; more integrated care with addiction specialists supporting non-specialists; and reducing the stigma experienced by people with SUDs.

## Background

“Things do not change; we change” (Henry D. Thoreau)*.* As Thoreau stated two centuries ago, the material world will not change, but after the pandemic, will humanity change? And what are the challenges we are facing in the treatment of substance use disorders (SUDs)? This short communication describes those challenges, and how to deal with them once the current pandemic declines, in the likely context of scarce resources.

### Current situation of COVID-19 and SUDs

The outbreak of severe acute respiratory syndrome coronavirus 2 (SARS-CoV-2) was considered a pandemic on March 11, 2020, by the World Health Organization. Many countries responded with physical distancing measures, reorganization of healthcare systems, lockdown regulations, and contingency plans for the economic situation [1].

Citizens affected by SUDs are high-risk populations for both SARS-CoV-2 infection and COVID-19-related mortality. Relevant vulnerabilities to COVID-19 in people who suffer SUD are described elsewhere (summary in Table 1).

**Table 1:** Summary of risk factors for COVID-19 in people affected by SUD

Substance-related factors [2–7]	Contextual and pattern of use related factors [8, 9]
• A protective effect of nicotine has been claimed, but a systematic review (5 studies, *n* = 1358) has shown that smoking is associated with adverse outcomes of COVID-19. Another study (169 hospitals, *n* = 8910) found the risk of death for current smokers during hospitalization due to COVID-19 79% higher than in non-smokers. • Alcohol use, especially heavy use, weakens the innate and acquired immune systems, thus increasing the risk of infections such as tuberculosis, HBV, HCV, HIV-AIDS, or COVID-19, and worsening the course of the disease. Heavy drinking is a well-established risk factor for acute respiratory distress syndrome. • Regular cannabis use is associated with coughing and other respiratory symptoms.	• Lung injuries related to vaping and/or smoking. • Lack of access to hand washing, disinfecting wipes, and personal protective equipment (PPE), and overcrowding among certain groups of people who use drugs (e.g., those in incarceration, homelessness). • Pulmonary hypertension associated with methamphetamine use. • Compromised immune function. • Stigma of drug and alcohol use as a barrier to accessing healthcare. • Sharing cigarettes, drinks, or needles is high-risk behaviors for becoming infected with SARS-CoV-2. • Lockdown has had an impact on illicit drug supplies, with a subsequent impact on behaviors of people who use substances.

Telemedicine for mental health problems including SUDs has taken a more central role during the crisis, showing its feasibility and minimizing the risk of virus transmission while providing continuity of care [10]. Telemedicine offers the promise of increased adherence to treatment, since a number of logistical barriers associated with physical attendance at treatment services are removed. In some countries (e.g., USA, Canada), confidentiality regulations and regulations requiring frequent in-person contact for those being treated with medication have been lifted during the public health emergency, thus making care more accessible and patient-centered than before. However, during this crisis, digital solutions have been hastily improvised and often lack support in the form of clinical guidelines for people with SUDs [11]. The addiction field has experienced a rapid increase of effective web-based teleconsultation platforms, which have created new methods of engaging with people with SUDs that are likely to continue after the crisis [12, 13]. Some specific groups (e.g., those with physical or cognitive comorbidities or health inequalities) may not be reached by these digital solutions, increasing the risk of social exclusion through the digital divide.

For the most severe cases, hospitalization at home has been a feasible solution [10]. Another solution has been designated COVID-19 wards or areas of outpatient treatment programs. Harm-reduction services have continued working in many countries (e.g., Canada, Spain, UK), but unfortunately, in other regions, they have had to reduce or pause their activities. The decrease in drug availability and consequences of this (e.g., withdrawal syndrome, reductions in purity) [9, 14] have led to rapid changes in clinical routines (e.g., larger take-home doses of methadone, reduced frequency of supervised consumption) [15]. Patients who have suffered from both SUDs and COVID-19 have been admitted to hospitals, requiring support from consultation-liaison psychiatry/addiction units due to pharmacological interactions, severe withdrawal syndromes including delirium tremens, craving management, or behavioral disturbances. Quarantine for homeless people who suffer SUDs has been organized through emergency shelters, which have unwittingly become an example of a “housing first” policy.

In summary, a range of new care approaches have been offered to people with SUDs during the COVID-19 pandemic, but many of these have been implemented hastily and need to be evaluated as we adapt to the next phases of the pandemic. New plans for care of people who suffer from SUDs in the “post-COVID-19 era” need to be developed, as we know this is a highly vulnerable group from economic, social, and health perspectives.

### The day after the pandemic and SUD treatment services

A global economic recession is expected [16], and its impact on drug use is currently unknown. We may expect decreased levels of substance use in the short term, due to decreased availability and affordability [17, 18]. However, the medium-term scenario is likely to be an increase in consumption for some populations as a consequence of increased emotional distress and psychiatric comorbidity [19, 20].

The COVID-19 pandemic and associated economic recession are causing isolation, unemployment, boredom, and emotional distress, factors that we know impact negatively and increase alcohol and other drug use [21–23]. Conversely, financial restrictions, reduced availability of illicit drugs, and higher prices of these during the lockdown have been well documented [24]. These factors have been shown to reduce alcohol and drug use, despite the fact that bars and cannabis social clubs reopened before schools and universities in some countries [25, 26]. In short, the existing literature identifies three factors which would tend to decrease substance use in this situation (reduced availability, higher prices, and financial restrictions) and three factors related to economic crises which could lead to increases in substance use (emotional distress, isolation, and unemployment).

Experiences from previous pandemics (e.g., SARS 2003) and natural or man-made disasters suggest a medium- and long-term increase in alcohol and drug use and consequent negative impacts after the pandemic [19].

Furthermore, the legal “drug” market (alcohol or tobacco) and “semi-legal drug” market (cannabis) will strive to maintain and even increase sales opportunities [27]. During the crisis, at least in high-income countries, some parts of the mainstream media and social media have encouraged people to drink when bored or to relieve stress [28]. In the US states where cannabis is legally available, medical cannabis shops have been deemed essential businesses, and there has been a surge in certification to receive medical cannabis. It is likely that this represents an increase in use which will in due course translate into incident cannabis use disorders and related harms.

As in the 2008 economic crisis, social and economic troubles will disproportionately affect those in the most vulnerable situations, including people with SUDs [20]. This population will request help not just for SUD but also for the consequences in other domains of health: especially in mental health, housing, and access to medications or other fundamental goods [17, 29]. Healthcare and social care systems need to prepare to cope with a likely increase in demand in this population.

A second concern is the competition for economic resources between SUDs and other disorders, and “new stigmatization.” Stigma is already an obstacle for treating people affected by SUDs [30]. In times of scarce resources, social stigma may influence how the allocation of economic resources is prioritized, leading to increased and systemic discrimination. Frontline healthcare staff and other at-risk populations will need mental health support after the pandemic [31]. Elective surgery and other interventions that have been put on hold during the pandemic will require increased resources to clear the backlog. In this scenario, governing politicians may be tempted to relegate SUD treatment to the end of the queue, even though this would be a “false economy,” as the problems are likely to be displaced onto already overstretched emergency services and acute hospitals [32].

## Main text

### How to prevent new stigmatization and even take advantage of the new opportunities: seven pillars

New clinical approaches implemented during the pandemic have helped to mitigate its impact on SUD treatment. But will they remain after the COVID-19 pandemic? We strongly advocate taking advantage of innovations initiated during the pandemic and continuing to develop them in a post-COVID-19 world. This situation is an opportunity to modernize SUD treatment, which is still largely based on that developed a century ago and only updated 40 years ago during the heroin pandemic. We believe that the renewed treatment systems should be based on the seven pillars described below. An overarching priority is to assure the renewal relies heavily if not exclusively on delivering treatments that have been proven to be effective, avoiding the temptation to use services that might seem to be readily available or that are promoted but that have no value.

Acceleration in the implementation of pillars 1 (telemedicine) and 2 (home hospitalizations) is necessary to cope with short-term and direct consequences of the pandemic, while pillars 3 to 7 are long-term and general measures to improve SUD treatment for the future.

#### Pillar 1: Telemedicine and digital solutions

Telemedicine in addiction shows favorable results for reduction of alcohol use and depressive symptoms; increased quality of life, patient satisfaction, and accessibility; and can be delivered at reduced cost [33]. Evidence for the efficacy of digital interventions is of good quality for alcohol and cannabis use disorders [34, 35]. During the COVID-19 pandemic, our anecdotal experience is that patients find such treatment more accessible and easier to adhere to. Some potential risks need systematic evaluation, such as larger prescriptions of opioid agonists, and less biological testing (e.g., urine drug testing) and clinical supervision. Additional technological solutions may also help to minimize these risks. In some countries, such as the USA, a proportion of the organizations were already in a better position to cope with the pandemic using telemedicine. A 2018 study found that 45% of 363 SUD organizations in the USA offered computerized screening and/or assessments, almost 30% provided telephone support or treatment, and 20% already offered video therapy [36]. Successful experiences with telemedicine in Italy, Spain, France, and the USA were reported during the current crisis, although these observations have not been backed up with specific clinical data [10, 37, 38].

However, use of technology in the SUD field is not just limited to online appointments. Smartphone and web-based interventions, text messaging for continuing contact and care, machine learning, and wearable devices, including digital phenotyping and ecological momentary assessment, biofeedback, and virtual reality, expand the range of available treatment opportunities and provide tools to help professionals and patients to make shared decisions [39, 40].

There is a need for a public health response to increase availability of treatment for SUDs, including training for healthcare professionals in online interventions and counseling. The concept of tele-expertise has also emerged as a new strategy that could be valuable in mitigating the impact of the pandemic on the SUD treatment system. In the tele-expertise framework, an addiction specialist could distantly supervise the work of other health professionals in the field [41].

#### Pillar 2: Home hospitalizations

Intensive outpatient treatments range in format from daily outpatient care in a hospital or center (e.g., day hospital) to home hospitalization. There are successful experiences in mental illness worldwide and emerging programs in SUD treatment [42–45].

Active healthcare treatment in the patient’s home (home hospitalization) is an alternative to inpatient treatment that has shown a reduction in readmissions, improvement of patient’s satisfaction, and reduction of hospital length of stay, with little or no differences in mortality for many medical conditions [46]. The evidence in the treatment of mental health disorders is more limited. Hospitalization at home has been implemented as an alternative for inpatient mental health treatment with encouraging results in both adults and children and adolescents [47, 48], and has shown feasibility during the COVID-19 crisis [10]. We strongly recommend that research and clinical practice of mental health home hospitalization include patients affected by SUDs. Furthermore, the link between telemedicine and home hospitalization may include nursing as daily contact and psychiatric and psychological support provided remotely.

In addition to home hospitalization, staying at home can also be viewed as “outpatient” treatment (e.g., where the patient is at home and has contact with a clinician electronically) and may for many people be preferable to inpatient treatment. Few if any studies find benefit for inpatient versus outpatient treatment for SUD, and thus, it is more appropriately reserved for patients who have no sober place to stay, lack social support or are vulnerable, need medical or psychiatric hospitalization, or have very severe SUD. During the pandemic, access to inpatient SUD care has been limited and greater use of outpatient treatment post-COVID-19 represents an advance in quality of care that should be maintained.

Since people living in deprived conditions are especially vulnerable to SUDs [49, 50], the implementation of pillars 1 and 2 should be carried out while in maintaining a focus on methods and processes which do not increase existing inequalities in treatment access.

#### Pillar 3: Consultation-liaison psychiatric and addiction services

Admission due to health problems in an acute hospital is an excellent opportunity to detect and manage SUDs, especially for patients with alcohol, opioid, and cocaine use disorders and multiple somatic and mental comorbidities [51]. Additionally, admission creates an opportunity for people with SUDs to access treatment at an earlier stage and close the treatment gap.

Multidisciplinary treatment facilitates the treatment of underlying conditions in many areas such as emergency departments, liver units, or head and neck surgery units [52]. It is also a helpful strategy to reduce stigmatization of people with SUDs and to promote an integrated treatment approach, with clinicians from different backgrounds (e.g., nursing, social workers, hepatologists, addiction specialists) working in an integrated way.

#### Pillar 4: Harm-reduction facilities

Abstinence is the most desirable objective for SUDs from a health perspective, but in some circumstances, this is not realistic (e.g., in severe and complex cases). Harm-reduction is a perfectly reasonable intermediate goal that many people can successfully achieve [53]. Harm-reduction aims and has been shown to reduce mortality and morbidity through a reduction in risk behaviors or a reduction of drug use, and is implemented in many countries because of its effectiveness [54]. “Housing first” is a practice for homeless people with co-occurring serious mental illness and SUD, which focuses on providing a stable home without requiring prior abstinence. This approach reduces homelessness and health service utilization and is efficient [55]. And, helpfully, albeit unwittingly, it has been used during the COVID-19 crisis to ensure the quarantine of this population.

#### Pillar 5: Person-centered care

Person-centered care is clearly preferred by patients, and advocated by many clinicians, but has not been widely implemented due to a combination of arcane regulatory structures and clinical inertia. During the acute phase of the pandemic, this has been put in place on an emergency basis. Patient preferences for fewer in-person visits and testing, easier access to enter into and receive treatment, and fewer prescribing restrictions are likely to have improved the quality of care and access to it. These should be retained post-COVID-19 as key elements of any new treatment system. Motivational interviewing (MI) and shared decision-making (SDM) are two effective approaches to SUD that have shown increasing evidence in the last 30 years [56, 57]. They have in common: (1) an ethical approach to SUD management, focusing on the self-determination principle (autonomy); (2) flexibility in objectives and treatment decisions; and (3) both remove the stigma of SUD. In other words, MI and SDM facilitate person-centered care.

#### Pillar 6: Promote paid work to improve quality of life in people with SUDs

Having meaningful paid work contributes significantly to both better outcomes and a reduction in healthcare costs for SUD patients, specifically (but not exclusively) for those with complex mental health comorbidities [58, 59]. In the aftermath of the COVID-19 pandemic, there have been many calls for future economic structures which lead to more sustainable economies, with a broader, socially inclusive scope [60]. We need to work together with these societal actors to include SUD patients in these new work contexts. The implementation of strategies such as “paid work or housing first” can be challenging in times of recession, when the forecast (from the International Labour Office) indicates that 6.7% of working hours will likely be lost during the second trimester of 2020 [61].

#### Pillar 7: Integrated addiction care

Reducing pressure and costs on acute and mental healthcare will be a priority after the pandemic due to increased demands on health systems. Cutting addiction services is a false economy as the impact is displaced onto already overstretched hospitals [32]. The solution is an integrated addiction care model that spans from early detection and brief interventions in primary care (both health and social) to highly specialized hospital services. Within this integrated approach, gambling and gaming problems deserve special attention.

The way to end the stigma experienced by these groups is to fully respect the civil rights of people with SUDs, including equality of access to health services. From the addiction treatment perspective, we have to guarantee access to evidence-based treatments in a modernized healthcare system, with these seven pillars offering a guide on how to do this and concurrently reduce stigma.

### Public health and prevention

Even though this communication focuses on SUDs and their treatment, in a time of crisis, it is also very important to implement preventive activities and take a public health perspective. Public health responses must be based on a realistic analysis of needs. The three “best buys” of the WHO (reduce availability, increase prices, and a ban on advertising) [62] and other evidence-based public health preventive strategies should be reinforced for legal drugs, including the alcohol, tobacco, and gambling markets (online and offline). In addition, new strategies must be implemented to systematically deal with (a) fake news concerning legal and illegal drugs and (b) controversial scientific information and messages (e.g., nicotine and protection from COVID-19)—especially when society is in a state of collective panic, people look for “the truth,” and science needs more time and greater integrity to provide clear answers.

The short-term impact of the pandemic has already been described, and relevant institutions (EMCDDA, AMSA, SAMSHA, and NIDA) have published guidelines on the management of this situation [8, 63–65]. This article focuses on the opportunities that could exist in the wake of the pandemic to make long-overdue improvements to the long-term healthcare of people who suffer from SUDs. We are fully aware that our world is facing a period of uncertainty and that this comes in a time that healthcare workers and systems are chronically overstretched. Nevertheless, we strongly feel the priorities outlined in the seven pillars should serve as a guide in the redesign of drug policies worldwide, irrespective of the different impacts and experiences of the COVID-19 outbreaks. Specifically, we think that the implementation of pillars 1 (telemedicine) and 2 (home hospitalizations) should be done quickly, as a short-term response to the pandemic, while pillars 3 to 7 represent much needed long-term and general measures to improve SUD treatment.

## Conclusions

The heroin pandemic four decades ago represents the most recent opportunity to update addiction treatment systems in many western countries. Now, the COVID-19 pandemic offers a unique opportunity to reshape and update addiction treatment networks, to properly face the challenges of the twenty-first century. This should include improved access to care, facilitated where appropriate by technology; more integrated care with addiction specialists supporting non-specialists; and reducing the stigma experienced by people with SUDs.

“Things MIGHT NOT change, but we MUST change”, at least to prevent the patients who suffer SUDs being last in the queue yet again.

## Data Availability

Not applicable

## Abbreviations

COVID-19: Coronavirus disease 2019
SARS-CoV-2: Severe acute respiratory syndrome coronavirus 2
SUDs: Substance use disorders
